# Darkfield-Confocal Microscopy detection of nanoscale particle internalization by human lung cells

**DOI:** 10.1186/1743-8977-8-2

**Published:** 2011-01-19

**Authors:** Eugene A Gibbs-Flournoy, Philip A Bromberg, Thomas PJ Hofer, James M Samet, Robert M Zucker

**Affiliations:** 1 Curriculum in Toxicology, University of North Carolina-Chapel Hill, Chapel Hill, NC, USA; 2 Center for Environmental Medicine, Asthma and Lung Biology, University of North Carolina-Chapel Hill, Chapel Hill, NC, USA; 3 Helmholtz Zentrum München, German Research Center for Environmental Health, Clinical Cooperation Group Inflammatory Lung Diseases, Gauting, Germany; 4 Environmental Public Health Division, NHEERL, U.S. EPA, Chapel Hill, NC, USA; 5 Toxicology Assessment Division, NHEERL, U.S. EPA, Research Triangle Park, NC, USA

## Abstract

**Background:**

Concerns over the health effects of nanomaterials in the environment have created a need for microscopy methods capable of examining the biological interactions of nanoparticles (NP). Unfortunately, NP are beyond the diffraction limit of resolution for conventional light microscopy (~200 nm). Fluorescence and electron microscopy techniques commonly used to examine NP interactions with biological substrates have drawbacks that limit their usefulness in toxicological investigation of NP. EM is labor intensive and slow, while fluorescence carries the risk of photobleaching the sample and has size resolution limits. In addition, many relevant particles lack intrinsic fluorescence and therefore can not be detected in this manner. To surmount these limitations, we evaluated the potential of a novel combination of darkfield and confocal laser scanning microscopy (DF-CLSM) for the efficient 3D detection of NP in human lung cells. The DF-CLSM approach utilizes the contrast enhancements of darkfield microscopy to detect objects below the diffraction limit of 200 nm based on their light scattering properties and interfaces it with the power of confocal microscopy to resolve objects in the z-plane.

**Results:**

Validation of the DF-CLSM method using fluorescent polystyrene beads demonstrated spatial colocalization of particle fluorescence (Confocal) and scattered transmitted light (Darkfield) along the X, Y, and Z axes. DF-CLSM imaging was able to detect and provide reasonable spatial locations of 27 nm TiO_2 _particles in relation to the stained nuclei of exposed BEAS 2B cells. Statistical analysis of particle proximity to cellular nuclei determined a significant difference between 5 min and 2 hr particle exposures suggesting a time-dependant internalization process.

**Conclusions:**

DF-CLSM microscopy is an alternative to current conventional light and electron microscopy methods that does not rely on particle fluorescence or contrast in electron density. DF-CLSM is especially well suited to the task of establishing the spatial localization of nanoparticles within cells, a critical topic in nanotoxicology. This technique has advantages to 2D darkfield microscopy as it visualizes nanoparticles in 3D using confocal microscopy. Use of this technique should aid toxicological studies related to observation of NP interactions with biological endpoints at cellular and subcellular levels.

## Background

The recent proliferation of nanotechnology combined with concerns over the health effects of human exposure to ambient ultrafine particulate matter (UFP) have created a need for information on the toxicology of nanomaterials. Studies to date have made it apparent that the effects of nanomaterials cannot be safely extrapolated from the toxicological properties of larger-scaled materials of the same composition [1,2].

Nano-scaled materials are generally defined as structures possessing at least one dimension that is 100 nm or less [3,4]. The small size and correspondingly large surface to mass ratio of nanomaterials are features which may alter their interactions with cells and tissues [5,6]. Incidental human exposure to environmental nanomaterials most often occurs through the inhalation of ambient ultrafine particulate matter that is primarily produced during the combustion of fossil fuels [7]. Conversely, nanomaterials that are intentionally engineered are more commonly known as nanoparticles. In this manuscript, the term nanoparticle (NP) will be used to refer to nano-scaled materials without regard to their origin and are considered to be under 100 nm in size.

Relative to ingestion and dermal absorption, inhalation of NP may be the most likely route of human exposure. The small size of NPs not only allows them to become airborne easily, but promotes deposition in the deep lung as well [1]. Indeed, inhaled UFP have been reported to be more potent in inducing adverse health effects than larger particles [1,3,8,9]. Some studies have suggested that inhaled NP penetrate the respiratory epithelial barrier and are distributed systemically to various organs and tissues, including the brain [1,5,10-12].

Imaging is a powerful technique for the study of cellular interactions with extracellular substrates, including particles [13,14]. Many critically important toxicological processes, such as the mechanisms through which nanomaterials penetrate into cells, are best addressed using imaging approaches. However, with the exception of fluorescently tagged synthetic particles, the small size of nanoparticles puts them beyond the limit of detection of about 200 nm using conventional bright-field light microscopy techniques. As an alternative, application of electron microscopy (EM) in NP studies has grown considerably in the past few years and remains the "gold standard" for many NP studies as this technology can easily observe particles below 100 nm in size. Unfortunately, EM is costly, labor intensive, limited to materials with sufficient electron density contrast, and primarily restricted to fixed specimens.

Conventional darkfield (DF) microscopy is an illumination technique used in light microscopy to optimize differences in contrast by selectively capturing light scattered by the specimen. In brief, this is accomplished with the attachment of a specialized light condenser that uses a light stop comprised of an annulus with a narrow aperture to obliquely illuminate the specimen via a hollow cone of light [15-17]. Using a confocal microscope, the light illuminating the specimen is focused by the objective, and collected by a darkfield condenser. Essentially, these instruments use a reverse light path from normal DF applications for DF detection in confocal mode. Consistent with normal DF illumination, the blockage of centralized light in DF-CLSM reveals only structures of the specimen that are capable of scattering light. DF detects light that is refracted, diffracted or reflected. This light scattering allows DF to detect extremely small structures, offering a potential light microscopy tool for the study of nanoparticles associated with cells. If a DF oil condenser with a 1.2-1.4 numerical aperture is used, it allows lenses with a higher numerical aperture to also be used which diminishes resolution losses in the microscope. However, the numerical aperture of the lens must be slightly below that of the numerical aperture of the condenser for DF to work. Previous studies have used DF imaging to observe NP [18-23]. Largely, these studies have been limited by visualizing NP in only a single 2D plane instead of producing a 3D image as presented in this communication.

In the present study, we show that the capability of DF to detect nanoparticles that can be effectively interfaced with a confocal laser scanning microscope (CLSM) to spatially localize nanoparticles along the z-axis of the cell, thereby permitting a determination as to whether nanoparticles are associated with the cell surface or within the cell. This communication reports the novel integration of DF and CLSM microscopy and its utilization in the measurement of nano-particle uptake and localization relative to intracellular organelles and the nucleus in cultured human lung cells.

## Methods

### Materials and Reagents

Green fluorescent 6.5 μm, 2.0 μm, 0.5 μm, 140 nm, 100 nm, and 50 nm polystyrene spheres (beads) were acquired from Polysciences Inc. (Warrington, PA) and Bangs Laboratories Inc. (Fishers, IN). Titanium dioxide nanoparticles with an average diameter of 27 nm were obtained from Degussa (Aeroxide TiO_2_, Parsippany, NJ). HCS Cell Mask Blue (H32720) and Prolong Gold Antifade Reagent with and without DAPI (P36934 and P36935) were purchased from Invitrogen (Molecular Probes, Eugene, OR). First Contact cleaning polymer was obtained from Photonic Cleaning Technologies (Platteville, WI).

### Specimen Preparation

#### Slide cleaning

A slide cleaning protocol was devised to minimize the presence of unwanted background debris detectable by darkfield microscopy [24,25]. New "pre-cleaned" microscope slides (Fisher Scientific, Pittsburgh, PA) were subjected to additional cleaning by wiping them using ammonia based glass cleaner and lens paper. As the slides were wiped, they were placed into a slide rack taking care to provide even spacing between each slide. Next, the slide rack was placed into a lidded vessel large enough to fully submerge the slides and washed successively in the following solutions: 1) 1:5 dilution of ammonia based glass cleaner in deionized water (dH20), 2) deionized water, and 3) 70% ethanol. Importantly, for each wash step, the lidded vessel containing the slides was placed in a bath sonicator and the entire apparatus was sonicated for 30 minutes in each wash solution. Washed slides were stored in 70% ethanol until needed. Just prior to coverslip mounting, a slide was removed from the 70% ethanol, briefly rinsed in 100% ethanol and allowed to air dry for approximately five minutes. Lastly, to further aid in the removal of slide debris, the specimen area of the slide was painted with a commercially available cleaning polymer (First Contact), allowed to cure for a minimum of 15 minutes, and peeled off immediately before mounting coverslips. All reagents used to wash slides were filtered using 0.22 μm filters. All steps in which the slides were exposed to air (i.e. removal from the various wash solutions) were carried out in a biological hood to minimize contamination with airborne debris. Use of this slide cleaning procedure produced slides that were relatively free of debris detectable by DF-CLSM.

#### Fluorescent Polystyrene Beads

Suspensions of 50 nm, 100 nm, 140 nm, 500 nm, 2 μm and 6.5 μm Fluorescent polystyrene beads (Polysciences, Warrington, PA) were prepared in dH_2_O and directly applied to freshly cleaned glass slides. Following a drying time of 15 to 20 minutes, #1.5 coverslips were mounted onto the slides using Prolong Gold Antifade Reagent.

### Cell Culture and Exposure

Transformed human airway epithelial cells (BEAS-2B, subclone S6; [26] were cultured as described previously [27] and maintained in serum-free KGM (Lonza, Walkersville, MD). The cells were incubated in a humidified incubator at 37°C in 5% CO_2_. For fixed cell studies, cells were sparsely plated (≤ 1.5 × 10^6 ^cells/well) on 12 mm, #1.5 coverslips located in 6-well culture dishes and allowed to grow for one day prior to exposure. At the time of exposure, media was removed from the cell cultures and 2 ml of a freshly prepared homogeneous suspension of 27 nm TiO_2 _was applied immediately. Cells were typically exposed for either 5 minutes or 2 hours (120 minutes) prior to washing and fixation. Pulsed exposures involved continuous exposure for 5 minutes followed by removal of exposure media, a brief wash using KGM, and incubation in fresh, particle free, media for an additional 115 min. After exposure, cells were washed twice in 1X Dulbecco's Phosphate Buffered Saline (PBS, Gibco, Grand Island, NY) and then fixed in 4% paraformaldehyde (PF) made up in PBS and stained.

#### Titanium dioxide Preparation

For each cell exposure experiment, a fresh stock solution of 27 nm TiO_2 _was prepared by resuspending 1 mg of dry particles in 1 ml of sterile dH_2_O. This solution was then sonicated for 30 seconds using a temperature-controlled cup-horn sonicator (Fisher Scientific, Pittsburgh, PA). The TiO_2 _particles were further diluted to their final concentration of 0.5 or 2.5 μg/ml using Keratinocyte Growth Medium (KGM). Just prior to cell exposure, these particle suspensions were sonicated again for 30 seconds and then immediately applied to 6-well dishes that contained 12 mm cover slips.

### Cell Fixation, Staining, and Mounting

Following NP exposure, cells were thoroughly washed in PBS, and fixed for approximately 30-60 minutes using 1 ml of 4% paraformaldehyde per well. After fixation, cells were stained using HCS Cell Mask Blue (CMB) as a cytoplasmic stain or 4',6-diamidino-2-phenylindole (DAPI) for nuclear staining. Staining using CMB was done as an adaptation of the procedure provided by the manufacturer. Briefly, 1 ml of 0.1 μg/ml CMB in 1× PBS was added to each well and allowed to incubate overnight at room temperature. The next morning, cells were washed in 1× PBS followed by a final rinse in dH_2_O. Lastly, the coverslips from each well were mounted on newly cleaned slides using Prolong Gold Antifade Reagent. In experiments where DAPI staining was performed, cells were mounted using Prolong Gold Antifade Reagent with DAPI in lieu of overnight staining.

### Confocal Microscopy Quality Assurance (QA)

The confocal microscope, lenses, and optical components utilized in this study were evaluated for QA using the procedures described by Zucker and Zucker et al. [28-32]. Briefly, colocalization was examined using PSF beads, and field illumination and laser powers were monitored. In the Nikon confocal system, laser fluorescence colocalization was present between the 488 and 561 lines, while the 404 showed a z axis spectral shift with the other 2 visible wavelengths (488 nm and 561 nm) and was not colocalized. This lack of colocalization of UV and visible laser light with the Nikon C1Si is quite typical of confocal microscopes from all manufacturers.

### Simultaneous DF and CLSM

**Figure 1:**
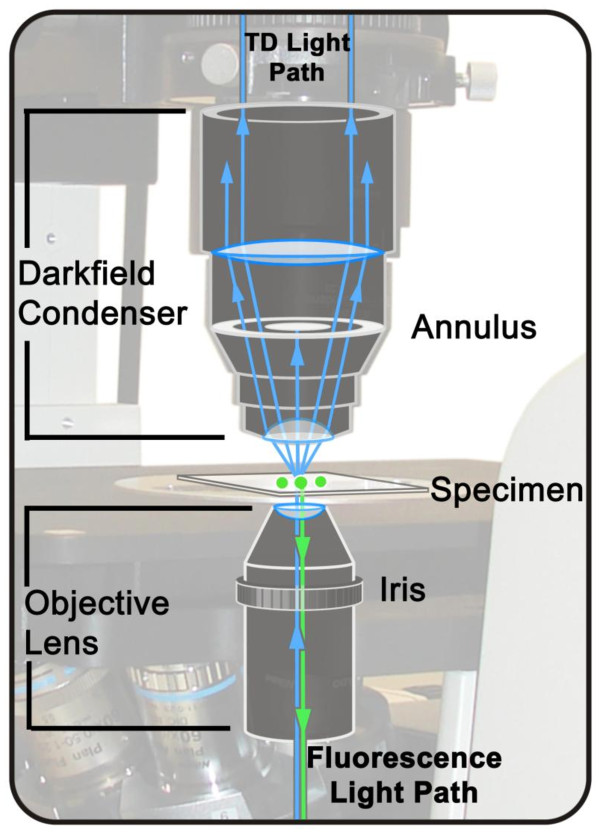
**Schematic diagram of an inverted confocal system equipped for simultaneous fluorescence and darkfield imaging**. In this example, source laser light (Blue) is focused on the specimen for fluorescence excitation. Fluorescence light emitted by the specimen (Green) is collected by the objective in a conventional CLSM manner. The darkfield condenser collects the scattered light. The numerical aperture of the objective is adjusted using the iris diaphragm to allow only excitation source light that is scattered by the specimen to reach the transmitted light detector (TD).

In conventional DF microscopy, light travels through a DF condenser with a numerical aperture that is higher than that of the objective lens. The light illuminates the sample and is detected by an objective with a lower numerical aperture than that of the condenser. It is useful to have an iris in the lens to accurately and ideally control the numerical aperture of the objective and the amount of scattered light entering it. In the technique described here the conventional DF optical path has been reversed. The light is focused on the sample with a lens containing an iris, and then the scattered light is detected with a transmitted light detector. The numerical aperture of the objective must be below the numerical aperture of the condenser for DF to work (Figure 1).

Imaging was carried out on two different configurations of confocal microscopes from Nikon and Leica. 1) A Nikon Eclipse C1si Spectral Confocal imaging system (Nikon Instruments Corporation, Melville, NY) equipped with an oil immersion Darkfield condenser (numerical aperture = 1.2 - 1.43) and 404 nm, 488 nm, 561 nm, and 633 nm lasers served as the primary means for experimental analysis. Specimens were observed via an Eclipse Ti microscope using either a 60X or 100X Plan Fluor oil immersion objective lens with adjustable iris permitting an numerical aperture ranging from 0.5 to 1.25. DF images were acquired via a transmission detector (TD). For fluorescence excitation, a conventional confocal optical path is maintained in which laser light originating from the objective lens is focused onto the specimen and then collected through the objective lens, Fluorescent light is then passed through an aperture (pinhole), and focused onto a detector consisting of a photomultiplier tube (PMT) tuned to detect specific wavelengths of light. Transmitted light detection of darkfield images begins with illumination by the same laser light originating from the objective lens. An occluding disc built into the darkfield condenser above the specimen blocks incoming light travelling on a direct path from the source but collects light scattered by the specimen and allows it to reach a transmission detector coupled to a PMT capable of monitoring multiple wavelengths of light. It is essential to have the numerical aperture of the condenser to be greater than the numerical aperture of the objective lens. 2) The 2^nd ^configuration was used to show the methodology is applicable on instrument from different manufacturers. A Leica DIRBE microscope with a dark field condenser, 1 mm condenser lens and Plan Apo 63x with an adjustable iris diaphragm that was variable between 1.32 numerical aperture and 0.6 numerical aperture was employed. This system used a dry condenser, compared with the oil condenser from the Nikon system. The Leica system used a Plan Apo lens with an air condenser while the Nikon system used a Plan Fluor lens with an oil condenser. A higher numerical aperture with better resolution was achievable with the Nikon system due to the oil condenser. However this oil condenser is not applicable on all manufacturers' inverted microscopes.

DF-CLSM images of fluorescent polystyrene beads observed in the absence of cells were acquired under 404 nm and 488 nm laser illumination/excitation while images of TiO_2 _exposed cells were obtained primarily using 404 nm laser light for better resolution. Cellular and organelle-specific fluorescent stains were used to image the space in which the NP were localized. Simultaneous fluorescent (Confocal) and transmission (Darkfield) imaging were performed for all two- and three-dimensional images acquired using this technique. All images were collected using Nikon EZ-C1 software, and post-acquisitional processing was performed using the Nikon NIS-Elements AR software package. The confocal slice number corresponding to maximal light scatter intensity was taken as the location of the nanoparticle. Similarly, the slice at which maximal fluorescence intensity was observed was used to identify the location of the nucleus. For statistical comparison, normalization of particle position relative to the nuclei of multiple cells was accomplished by subtracting the nuclear slice number from the particle slice number, dividing by the total number of slices, and multiplying by a factor of 100 to obtain the closest whole-number.

### Statistical Analyses

Pairwise comparisons were analyzed by the Wilcoxon's signed rank test using GraphPad Prism statistical software (San Diego, CA); p < 0.05 was considered significant.

## Results

### DF-CLSM detection of nanoscale particles

**Figure 2:**
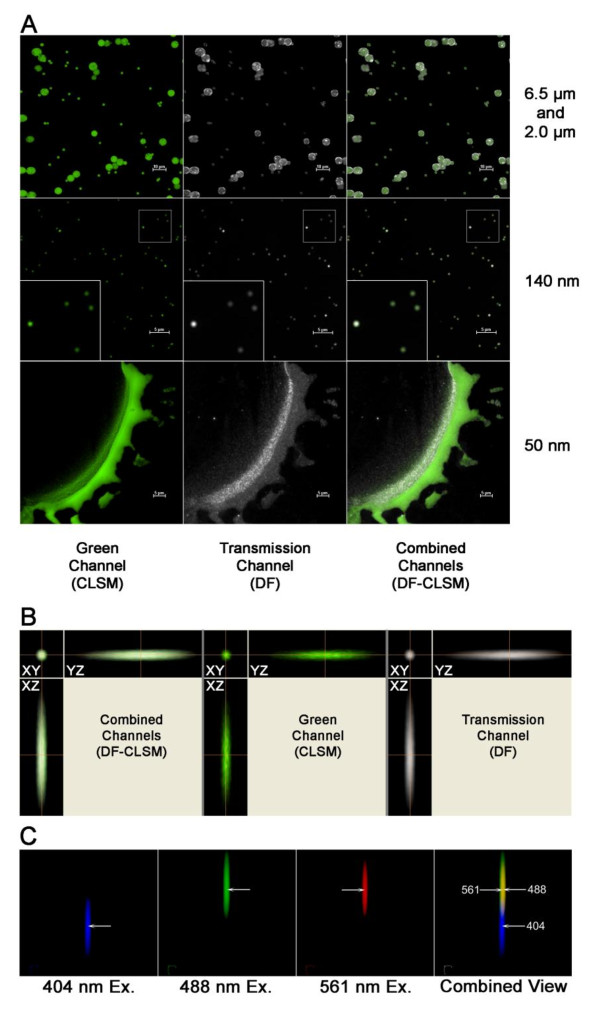
**Detection of fluorescent polystyrene spheres by co-localized confocal and darkfield microscopy**. A. Fluorescent polystyrene beads of the indicated sizes were illuminated with 488 nm laser light. Emitted particle fluorescence was detected using the confocal microscope in the green channel while scattered incident light collected by the darkfield condenser was simultaneously observed via the transmitted light detector. For the 140 nm spheres, insets in the lower left-hand corner show an enlarged area for clarity. B. Three dimensional colocalization analysis of a 500 nm fluorescent polystyrene sphere imaged by DF-CLSM. The sphere shown was excited by 488 nm laser light while simultaneous monitoring for fluorescence and scattered light occurred via the green and transmitted light channels. Each set of images shows the XY, XZ, and YZ orientation for the combined, green (CLSM), and transmission (DF) channels, respectively. The large crosshairs represent the same point in space across all the axial views. C. Variability in spatial localization of DF images obtained with multiple wavelengths of light. Shown are 10x pseudo-colored images of the same 27 nm TiO_2 _particle illuminated by 404 (Blue), 488 (Green), and 561 (Red) laser light. Each set of images depicts the X, Y, and Z orientation for the transmission (DF) channel. For the combined view, areas of overlap in the 488 and 561 excitations are observed in yellow. The arrows represent the midpoint of the same particle illuminated at each wavelength, showing a small x,y lateral distortion between the 488 and 561 nm excitation lines, while both the 488 and 561 have a much larger axial (z) dispersions from the 404 excitation line. Note the large separation between the blue exciation and the visible laser exciation lines. 60x Plan Fluor, Magnification 600X + 1, 5, or 10X zoom as designated.

In order to validate the DF-CLSM approach to nanoparticle detection, we imaged various intrinsically fluorescent polystyrene spheres of known size, ranging from 50 nm to 6.5 μm in diameter. As demonstrated in Figure 2A, the DF and CLSM images of polystyrene spheres colocalized in the XY plane with discrete particles down to 100 nm in diameter using the 488 nm laser line for optimal fluorescence excitation. Preparations of 50 nm fluorescent particles showed weak fluorescence with 488 nm excitation, but could still be resolved as monomeric units by DF detection (Figure 2A). Figure 2B confirms colocalization of the DF particle image with the fluorescence image in all three spatial axes. These results demonstrated the feasibility of the DF-CLSM combination in detecting particles in the nano size range using a transmitted light detector and correlating these transmitted light images (DF) to a specific fluorescence signal which was derived from the same laser source. Experiments using 3 laser lines revealed that colocalization of the 404 nm, 488 nm and 561 nm excitation wavelengths was not attainable in the DF-CLSM optical path (Figure 2C). There was a z-axis distortion between 404 nm and remaining visible laser lines, as well as a lateral shift between the 561 nm and 488 nm excitation lines. The co-localization problems were greater with NP than it was with micron and submicron particles. The use of multiline lasers including the 404 nm laser will yield significant axial distortion in the cellular localization of the particle. For these reasons, subsequent measurements made with DF-CLSM were restricted to single-line excitation or sequential multi-line acquisitions in the visible range with only one laser line being used to excite the DF signal. As expected the shortest wavelength (404 nm) was found to provide the best resolution and was used for high resolution work. However the 488 laser provides more functionality as it is compatible with a greater number of cellular probes.

**Figure 3:**
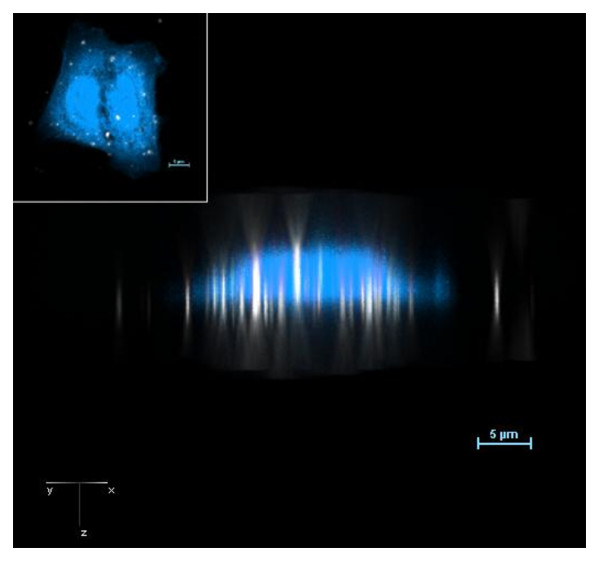
**Darkfield(DF)/Confocal(CLSM) imaging of TiO_2 _nano particles internalized by human bronchial epithelial cells**. BEAS 2B cells were exposed to 27 nm TiO_2 _for 2 hours followed by fixation and staining with HCS Cell Mask-Blue to visualize the cytoplasm. Shown is a pseudo-colored side view taken along the Z-axis of these cells. Particles appear as elongated structures due to the lack of confocality in the darkfield optical path. The inset displays a top (XY) view of the same cells. Cells were excited using only the 404 laser line while fluorescence was monitored using the blue channel. Magnification 1800x with a 3X zoom. 60x plan fluor objective

We next evaluated the utility of the DF-CLSM approach in detecting nanoparticles associated with cells. BEAS 2B cells were exposed to 27 nm TiO_2 _for 2 hrs, fixed and imaged using DF-CLSM. Three-dimensional analysis of these cells stained with Cell Mask Blue (CMB) revealed the expected cellular morphology (Figure 3). Due to the lack of confocality, and the scattering of light in the DF light path, three-dimensional reconstruction depicts nanoparticles as elongated rod-like structures whose longitudinal spread extends symmetrically through the slices of the z-stack. A typical fluorescent PSF (Point Spread Function) is not obtained in darkfield with the DF-CLSM method. When superimposed on the confocal image of the cell, the TiO_2 _nanoparticle rods appeared to localize through the body of the cell, suggesting that they are within the cells. The center of the nanoparticle can be determined by the position of maximum intensity in the cigar-shaped image of the NP.

### Examination of in vitro particle internalization using DF-CLSM

**Figure 4:**
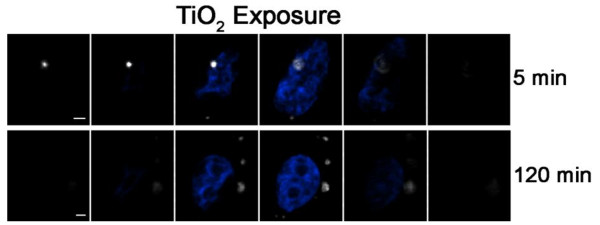
**Image galleries of BEAS cells exposed to nanoparticles for 5 and 120 min**. Shown are optical sections taken from a confocal z-stack of nuclear fluorescence with darkfield detection of NP. Results show time-dependent internalization of nanoparticle uptake by human bronchial epithelial cells imaged by Darkfield (DF)/Confocal (CLSM) microscopy. BEAS-2B cells were exposed to 27 nm TiO_2 _particles for 5 min (top gallery) or 120 min (bottom gallery), fixed and stained with DAPI. The nanoparticles (depicted as white) were located in slices above the blue nucleus at 5 min but were on the same sections as the nuclei at 120 min. Cells were excited using the 404 laser line while fluorescence was monitored using the blue channel. Scale bar represents 2 μm. Magnification 3000x with a 5X zoom.

We next undertook a series of experiments designed to measure particle internalization using DF-CLSM by exploiting the expectation that particle internalization is a time-dependent process. Figure 4 shows abridged galleries corresponding to representative slices from z-stack images for BEAS 2B cells exposed to 27 nm TiO_2 _for 5 or 120 min. After incubation, the cells were washed in PBS and fixed in a medium containing DAPI to stain the cell nucleus. Relative to the 5 min time point, nanoparticles in cells exposed to TiO_2 _particles for 120 min were detected at slice numbers corresponding to deeper locations within the cell (Figure 4), consistent with a time-dependent penetration of particles into the cell. The actual position of the particle can be determined by the confocal slice that has the maximum intensity in the DF image.

**Figure 5:**
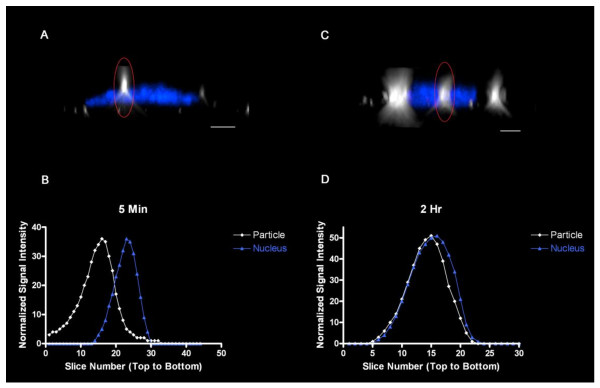
**Determination of nanoparticle location using the maximum intensity technique**. Scatter and fluorescence intensity plots through the z-axis of the entire cell volume are plotted for cells incubated with TiO_2 _for 5 min and 2 hr time points for both a selected particle of interest (circled in red) and the corresponding nucleus, respectively. Fluorescent and scatter light intensity values have been normalized to the same peak height for clarity. Scale bar represents 10 μm. Magnification 3000x with a 5X zoom.

In order to provide an empirical expression of nanoparticle penetration in the cell, scattered light and DAPI fluorescence intensities were plotted as a function of z-stack slice number for BEAS cells exposed to TiO_2 _for 5 or 120 min (Figure 5). Following 5 min of exposure, the peak scatter intensity of a representative particle was found at a slice number located above the slice that corresponded with the peak fluorescence of the nucleus (Figure 5, panels A and B). In contrast, by 120 min of TiO_2 _nanoparticle exposure, the peak scatter intensity of a representative particle was found at a slice number that coincided with the location of the center of the nucleus (Figure 5, panels C and D). This shows that this technique can be used to measure the transport of particles though the cell.

**Figure 6:**
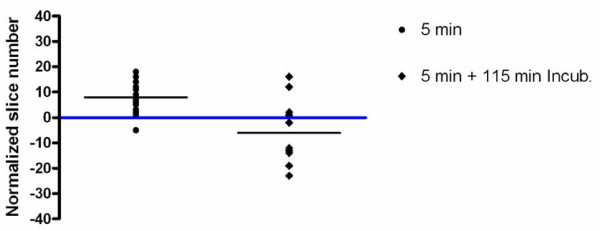
**Statistical analysis of mean particle location in cells exposed to 27 nm TiO_2 _for varying lengths of time**. Particle localization within the z-axis was taken as the optical section containing maximal intensity in the dark field channel, and is plotted relative to the section in which DAPI fluorescence was most intense (center of nucleus normalized to zero, depicted as horizontal blue bar). Within each group of particles, the black line illustrates an averaged (mean) particle location for the center of each particle, as detected using DF-CLSM. Note that at 120 min incubation the particles are located above, below and at the midpoint of the nucleus, suggesting variations in cell morphology and distinct transit paths.

To further test this approach in a practical application, we compared the location of maximal particle scatter intensity relative to the nucleus in the z-axis for BEAS cells exposed to 27 nm TiO_2 _continuously for either 5 min, or "pulsed" for 5 min followed by washing and an additional 115 min incubation in particle-free media prior to fixation (120 min-pulsed). The data were normalized for slice number and the slice in which the nucleus was centered was designated as zero. Similar to the results in Figure 5, a clear shift in the mean particle location inferred from maximal light scatter was observed between cells exposed to particles for the 5 and the 120 min-pulsed groups. Specifically, there was a time-dependent change in the location of the particle maximum intensity and presumed center away from the cell surface towards the nucleus and slide surface (Figure 6). Interestingly, the 120 min-pulse cells showed particles clustered above, coincident with and below the nucleus center,. This may reflect distinct intracellular paths established by the presence of the nucleus within a large fraction of the cell volume or may be due to in part to the variation in intracellular "z" volumes. These findings demonstrate that DF-CLSM is useful to observe time-dependent NP transit within the cell.

## Discussion

While the use of NP in consumer products, industrial processes, and pharmaceutical applications continues to grow, knowledge of the impact of nanomaterials on human health remains limited [33]. Compounding the challenge presented by the paucity of toxicological information on nanomaterials, investigators studying the biological effects of nanomaterials are faced with a number of unique challenges. Fundamental questions pertinent to the interaction between nanoparticles and the cell, are best addressed with imaging studies. However, NP are usually not detectable by conventional light microscopy methods. In some instances, fluorescent nano materials (i.e., Q-dots) can be used with fluorescent microscopy. Darkfield microscopy can detect the presence of nanoparticles, but their location within the cell or on the surface of the cell can not be determined with accuracy using a wide field microscope. The combination of darkfield and confocal microscopy described in this manuscript was developed in an effort to address some of these imaging challenges by bringing the strengths of two distinct light microscopy techniques (DF and CLSM) to study the interactions of cells with environmentally relevant NP.

While confocal and wide-field fluorescence microscopy are commonly used to image synthetic nanoparticles tagged with fluorophores (e.g., Q-dots) evidence that particle surface chemistry plays a critical role in toxicity raises questions about the relevance of these materials as surrogates for "real world" nanoparticles [34,35]. Electron microscopy (EM) is usually thought of as the "gold standard" for investigation of the biological impacts of nanomaterials. Unfortunately, EM can be quite laborious and costly to employ. Darkfield microscopy is essentially an illumination-based imaging technique used to enhance the contrast of specimens for increased visibility [15-17,36]. Confocal microscopy is a multifaceted light microscopy technique that contributes increased resolution and optical sectioning of specimens for three dimensional analyses, as well as the precise application of excitation wavelengths [37-39]. The utility of DF-CLSM in detecting NP was established by experiments in which we show that the DF scatter signal colocalizes with the fluorescence of polystyrene particles with sizes as low as 50 nm in diameter. In fact, the DF signal appeared to provide superior discrimination of monomeric units relative to fluorescence for 50 nm particles. In this study, the location of maximal scatter intensity in the z-axis was presumed to be the physical center of the particle. Using this approach, it was possible to determine the depth of penetration of the particle within the cell relative to an organelle or the nucleus. In separate experiments, similar measurements of particle penetration were made using DF-CLSM with the cell membrane as a reference (Gibbs, unpublished). Thus, the combination of DF optics with the power of a confocal microscope yields an image in which NP can be visualized within a narrow and precise focal plane for the spatial determination of NP within the cell in the z-axis. With the application of currently available and novel deconvolution processing algorithms, it may be possible to better localize the NP within the volume of the cell in three dimensions. Importantly, the DF-CLSM technique allows the visualization of non-fluorescent particles, making it suitable to the study of environmentally relevant NP. An additional advantage of DF-CLSM is its potential to be used in experiments involving live-cell imaging, which is impossible with conventional EM.

Detection of NP using DF-CLSM relies heavily on the intrinsic light scattering properties of the material being observed. In the validation of this technique, DF-CLSM proved to be sufficiently robust to detect several types of NP comprised of different materials ranging from polymers to nanodiamonds and metal oxides (Gibbs et al, unpublished). Interestingly, even materials with relatively smooth surfaces, like the polymers of polystyrene beads, have sufficient light scattering properties to be detected by this technique. The associated signal from larger polystyrene beads is less than that produced by smaller TiO_2 _nanoparticles.

The DF-CLSM imaging approach holds considerable promise for future applications in the study of the biological interactions of NP. While in the present study we chose to use a simple nuclear stain in the assessment of particle internalization, other biological processes and interactions could be examined by DF-CLSM using the numerous biosensors and staining reagents available. For laboratories with existing CLSM imaging equipment, implementation of DF-CLSM requires only the acquisition of a relatively inexpensive darkfield condenser and objective lenses with a suitable variable iris to adjust the numerical aperture, or objectives lenses with low fixed numerical aperture values. We have been able to successfully adapt both Leica and Nikon inverted confocal microscopes with condensers costing between $70 and $700, respectively.

As with any technique, DF-CLSM has limitations. In this regard, it is important to bear in mind that this application of DF is a detection method, not a technique that permits direct observation or exact size of the NP. Thus, this method does not produce an accurate size representation of nanoparticles. Likewise, agglomerates of nanoparticles may be represented as a single point with a larger size and increased light scatter. Therefore, inferences regarding size, shape, and number (i.e., agglomeration) of the structures being examined are limited, as other factors such as surface irregularity and reflectivity contribute significantly to the signal strength in DF detection. Furthermore, although they are less expensive than their high numerical aperture counterparts, it should also be noted that low numerical aperture objectives required for DF present disadvantages as well. Low numerical aperture objectives impose limits on optical resolution and may introduce chromatic aberration into the image. In our characterization of this technique, we have found that choosing a single excitation wavelength is the best way to avoid chromatic aberration and colocalization errors. Similarly, the DF and confocal fluorescence signals should ideally be acquired using the same excitation wavelength in order to avoid possible colocalization errors between different wavelengths of lights, as shown in Figure 2C. In cases where multiple excitation wavelengths are needed, they should be used sequentially rather than simultaneously and the 404 nm line should not be used with the visible wavelenghts. By default the shortest wavelength should be used for the acquisition of the DF signals, but it is important to use a wavelength in which the NP is being localized with the fluorescence structure. Notably, appropriate use of the excitation wavelengths used during image acquisition must be taken into account because data acquired with sequential excitation of multiple wavelengths may introduce chromatic aberration errors in the sample between the nanoparticles derived from one wavelength and the fluorescent signals derived from the other laser lines. This is not necessarily a limitation unique to this application, but more likely a limitation resulting from the UV and visible colocalization issues in CLSM and the quality and alignment of the optical components used in the CLSM equipment. It appears that the nanoparticles accentuate these colocalization problems relative to submicron and micron particles. It is recommended that characterization of issues regarding chromatic aberration and colocalization of various wavelengths should be made for each imaging system and each lens used.

## Conclusions

Even with the acknowledged limitations, the DF-CLSM methodology represents a novel and workable option for many investigators searching for a light microscopy technique that can be used to study the interactions between cells and NP in the environment. By interfacing the ability of darkfield microscopy to use light scatter to detect very small structures with the power of confocal microscopy to render specimens in three dimensions, DF-CSLM provides a solution to the unique challenges of conducting toxicological studies in the nano-scale. The DF-CLSM technique is superior to widefield DF as it uses the power of the confocal microscope to produce 3D images of nanoparticles within cells.

## Competing interests

The authors declare that they have no competing interests.

## Authors' contributions

EGF conducted most of the experiments and was the lead writer. All authors contributed to the experimental design, interpretation of the data and preparation of the manuscript. All authors read and approved the final manuscript.
